# Screening for IgG4-type anti-nuclear antibodies in IgG4-related disease

**DOI:** 10.1186/s12891-015-0584-4

**Published:** 2015-05-28

**Authors:** Kazuhiro Kiyama, Hajime Yoshifuji, Tsugumitsu Kandou, Yuji Hosono, Koji Kitagori, Ran Nakashima, Yoshitaka Imura, Naoichiro Yukawa, Koichiro Ohmura, Takao Fujii, Daisuke Kawabata, Tsuneyo Mimori

**Affiliations:** Department of Rheumatology and Clinical Immunology, Graduate School of Medicine, Kyoto University, 54 Shogoin-Kawahara-cho, Sakyo-ku, Kyoto, 606-8507 Japan

**Keywords:** IgG4-related disease, Systemic autoimmune disease, IgG subclass, Autoantibody, Anti-nuclear antibody

## Abstract

**Background:**

Immunoglobulin (Ig) G4-related disease (IgG4-RD) is characterized by elevated serum IgG4 and infiltration of IgG4^+^ plasma cells into multiple organs. It is not known whether serum IgG4 is autoreactive in IgG4-RD.

**Methods:**

We measured anti-nuclear antibody (ANA) in 19 IgG4-RD cases, determined IgG subclasses of the ANA, and compared them with those of other systemic autoimmune diseases (systemic lupus erythematosus, Sjögren’s syndrome, systemic sclerosis, and polymyositis), using subclass-based ANA test (indirect immunofluorescence).

**Results:**

58 % of IgG4-RD cases were ANA-positive (cut-off: 1:40). Whereas their subclass of ANA was predominantly IgG2, we observed no IgG4-type ANA. In systemic autoimmune diseases, subclasses of ANA were mostly IgG1, 2, or 3, but IgG4-type ANA was very rarely detected. We also found several patients in whose serum ANA patterns differed among IgG subclasses, probably due to the difference of corresponding autoantigens.

**Conclusions:**

Although IgG4 is highly elevated in sera of IgG4-RD patients, their ANA do not include IgG4 subclass. These results offer new insight into the role of IgG4 and the pathogenesis of IgG4-RD, implying that each IgG subclass tends to cover its own spectrum of antigens, and IgG4 is not preferentially used to make ANA.

## Background

Immunoglobulin (Ig) G4-related disease (IgG4-RD) is a multi-organ disorder characterized by elevated serum IgG4, organ infiltration by IgG4^+^ plasma cells, hypergammaglobulinemia, and tissue sclerosis [1–4]. Many organs, such as lacrimal gland, salivary gland, eye orbit, lymph node, thyroid gland, lung, pancreas, kidney, retroperitoneum, and prostate can be affected by IgG4-RD. The role of IgG4 in IgG4-RD is not sufficiently understood. Some view IgG4-RD as an allergic disease, because IgG4-RD is often complicated in allergic diseases and serum IgE levels are often high in IgG4-RD. Others see IgG4-RD as an autoimmune disease, because anti-lactoferrin [5] and carbonic anhydrase II [6] antibodies are detected in some of IgG4-related autoimmune pancreatitis cases, and because IgG4-RD cases usually show good responses to glucocorticoid therapies.

At this point, there is no consensus that IgG4-related disease is an autoimmune disorder. To examine whether IgG4 in IgG4-RD is autoreactive, we determined IgG subclasses of serum anti-nuclear antibody (ANA) in IgG4-RD patients and compared them with those in patients with systemic autoimmune diseases such as systemic lupus erythematosus (SLE), Sjögren’s syndrome (SS), systemic sclerosis (SSc), and polymyositis (PM). Using a subclass-based ANA test that was derived from indirect immunofluorescence (IIF), we investigated how frequently IgG4 was included in ANA in IgG4-RD. We also examined how frequently each IgG subclass was included in ANA in systemic autoimmune diseases.

## Methods

### Patients

Patients were recruited from Department of Rheumatology and Clinical Immunology, Kyoto University Hospital, Kyoto, Japan. The patients were definitely diagnosed by the 2011 Comprehensive Diagnostic Criteria proposed by the IgG4-RD research team of Ministry of Health, Labour and Welfare (MHLW), Japan [4]: (1) diffuse or localized swelling or mass formation of ≥ 1 organs, (2) elevated serum IgG4 levels ≥135 mg/dL, (3a) fibrosis with remarkable infiltration of lymphocytes and plasma cells, and (3b) IgG4^+^/IgG^+^ plasma cell ratio > 0.4, and > 10 IgG4^+^ plasma cells in a high-power field. No IgG4-RD patients were considered having SS, Castleman’s disease, sarcoidosis, granulomatosis with polyangiitis, or malignant lymphoma. As ANA-positive disease controls, we enrolled 8 SLE patients diagnosed by the 1997 American College of Rheumatology revised criteria [7], 8 SS patients diagnosed by the 1999 revised criteria of MHLW, Japan [8], 4 SSc patients diagnosed by the 1980 American College of Rheumatology criteria [9], and 7 PM patients diagnosed by Bohan and Peter’s criteria [10]. All participants provided informed consent in accordance with the Declaration of Helsinki. This study was approved by the Medical Ethics Committee of Graduate School of Medicine and Faculty of Medicine, Kyoto University.

### Detection of subclass-specific ANA

We performed subclass-based ANA tests based on the Fluoro-HepANA™ test (Medical & Biological Laboratories, Nagoya, Japan). Briefly, HEp-2 cell-coated slides were incubated with sera, washed with PBS, incubated with FITC-labeled second antibodies, and observed with a fluorescence microscope. Instead of using anti-total human IgG antibody as the second antibody, we used anti-IgG1 (ab50473, Abcam), anti-IgG2 (10122, Alpha Diagnostic Intl.), anti-IgG3 (10123, Alpha Diagnostic Intl.), or anti-IgG4 antibodies (ab99821, Abcam). To detect total-IgG ANA, patients’ sera are usually diluted by the ratios starting from 1:40. To detect each IgG-subclass ANA, the sera were not diluted because of relatively low affinities of the second antibodies against subclasses.

## Results

### ANA positivity of IgG4-RD

Of 19 cases that definitely satisfied the 2011 Comprehensive Diagnostic Criteria for IgG4-RD by MHLW, Japan (Table 1), 14 (74 %) were older than 60 years, and 14 (74 %) were male. Lymph node swellings and retroperitoneal fibrosis were major manifestations. Eleven patients (58 %) were ANA-positive at a cut-off titer of 1:40 (range: 1:40–1:320). The ANA patterns were homogeneous + speckled or speckled in most cases. Although 7 (37 %) were positive for rheumatoid factor and 2 (11 %) were positive for anti-SS-A/Ro antibodies, we confirmed these 9 cases did not fulfill the criteria for rheumatoid arthritis (RA) or SS. No patients were positive for anti-DNA, Sm, or U1-RNP antibodies.

**Table 1:** Clinical, serological, and histopathological features of IgG4-RD cases

Case	Age	IgG4^a^	IgG^a^	ANA Specific Abs	RF^b^	Clinical manifestations	Biopsy source, IgG4^+^/IgG^+^ cell ratio
1	73	2890	3668	40 (Homo + Spe)	<6	Mikulicz’s disease, Prostatitis, LN	Prostate, 0.60
2	76	2210	3632	40 (Spe)	<6	Mikulicz’s disease, RPF	Submandibular gl, 0.40
3^c^	79	1460	3669	160 (Homo + Spe) Anti-SS-A^+^	<6	Küttner’s tumor, IP, IN, RPF, LN	Submandibular gl, 0.73
4	66	1090	2301	40 (Homo + Spe)	30.3	AIP, IN, Renal pseudotumor	Kidney, 0.70
5^c^	73	592	3321	320 (Homo + Spe)	<6	Sialadenitis, IP, IN, RPF, LN	Submandibular gl, 0.43
6	74	389	2184	<40	<6	Retroorbital tumor	Retroorbital tumor, 0.48
7	52	383	1748	<40	<6	Küttner’s tumor	Submandibular gl, 0.57
8	70	724	1729	<40	<6	Küttner’s tumor, LN	Submandibular gl, 0.40
9	46	675	1617	80 (Homo + Spe)	26.8	Mikulicz’s disease	Lachrymal gl, 0.41
10	37	533	1741	<40	<6	Mikulicz’s disease	Lachrymal gl, 0.50
11	76	458	1527	<40	<6	AIP, RPF	Retroperitoneal tumor, 0.70
12	62	315	1809	40 (Spe) Anti-SS-A^+^	<6	AIP, RPF	Pancreas, 0.43
13	79	1960	2953	40 (Homo + Spe)	65	Orbital tumor, Lung nodule, LN	Orbital tumor, 0.59
14^c^	62	1460	2177	40 (Spe)	23.3	Sialadenitis, Laryngeal tumor, LN	Parotid gl, 0.60 Cervical LN, 0.69
15	65	1050	1811	<40	19.8	Mikulicz’s disease, LN	Submandibular LN, 0.80
16	25	1210	2181	<40	<6	Mikulicz’s disease, IP, IN, Renal pseudotumor, LN	Minor salivary gl, 0.65
17	55	1510	3116	<40	72.2	Orbital tumor, RPF, Lung nodule, LN	Cervical LN, 0.90
18	61	491	1466	80 (Spe + Granular)	<6	Sialadenitis	Submandibular gl, 0.48
19	78	1470	3762	80 (Homo + Spe)	35	AIP, RPF	Vater’s ampulla, 0.48

^a^mg/dL in serum. ^b^IU/mL. ^c^Shown in Fig. 1

ANA: anti-nuclear antibody; gl: gland; Homo: homogeneous; IN: interstitial nephritis; IP: interstitial pneumonitis; LN: lymph node; RF: rheumatoid factor; RPF: retroperitoneal fibrosis; Spe: speckled

### IgG subclasses of ANA in IgG4-RD

**Fig. 1:**
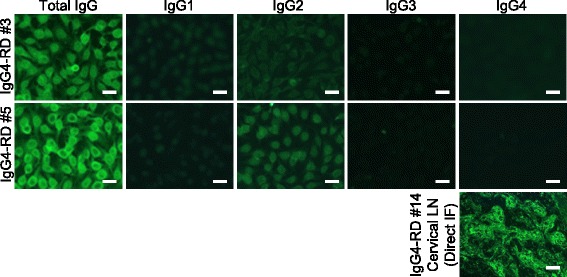
Subclass-based ANA test in IgG4-RD, showing immunofluorescence microscopy of two typical IgG4-RD cases (IgG4-RD #3 and #5). Lower right panel: We confirmed the second antibody’s function by direct immunofluorescence of a lymph node specimen (IgG4^+^/IgG^+^ plasma cell ratio = 0.69) from an IgG4-RD patient (IgG4-RD #14). Bar = 20 μm

**Fig. 2:**
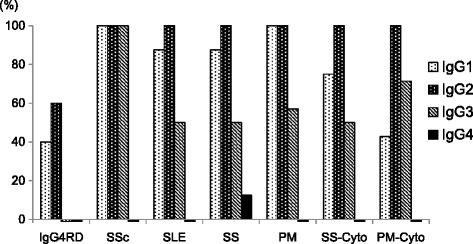
Positivity of each ANA subclass in IgG4-RD and systemic autoimmune diseases. Cyto: positivity of each subclass of anti-cytoplasmic antibody was also calculated for patients with Sjögren’s syndrome and polymyositis

We selected 5 IgG4-RD patients robustly ANA-positive with a cut-off titer of 1:80, and examined the IgG subclasses of their ANA. Subclass-based ANA test showed IgG2^+^ ANA and scant IgG1^+^ ANA. However, we found no IgG4^+^ or IgG3^+^ ANA (Fig. 1, 2). We confirmed that the second antibody against IgG4 worked, using direct immunofluorescence on a lymph node specimen (IgG4^+^/IgG^+^ plasma cell ratio = 0.69) of an IgG4-RD patient (Fig. 1, lower right panel).

### IgG subclasses of ANA in systemic autoimmune diseases

**Fig. 3:**
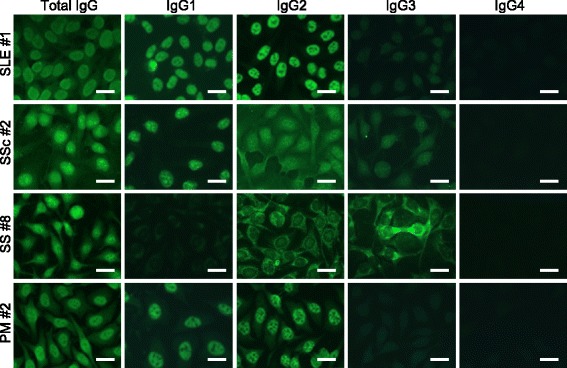
Subclass-based ANA test for systemic autoimmune diseases showing immunofluorescence microscopy for each typical case, including systemic lupus erythematosus (SLE #1), systemic sclerosis (SSc #2), Sjögren’s syndrome (SS #8) and polymyositis (PM #2) showed variation in ANA patterns among IgG subclasses. In SSc #2, total IgG showed Discrete spe + Speckled + Cyto, while IgG1 showed Discrete spe + Speckled, IgG2 showed Discrete spe + Speckled + Cyto, IgG3 showed Discrete spe + Cyto, and IgG4 showed negative. In SS #8, total IgG showed Speckled + Nucleolar + Cyto, while IgG1 and IgG2 showed Speckled + Cyto, IgG3 showed Nucleolar + Cyto, and IgG4 showed negative. Bar = 20 μm Discrete spe: discrete speckled, Cyto: cytoplasmic

**Fig. 4:**
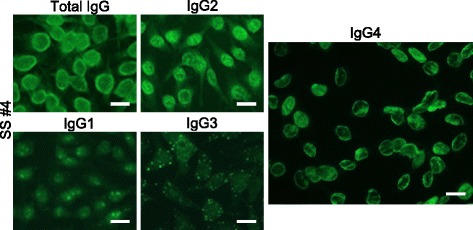
Subclass-based ANA test of a patient with Sjögren’s syndrome (SS #4) showing IgG4-type ANA. ANA patterns differed among IgG subclasses. Total IgG showed Speckled, while IgG2 showed Speckled + Cyto, IgG1 and IgG3 showed Nucleolar + Cyto (with atypical cytoplasmic spots), and IgG4 showed Peripheral. Bar = 20 μm. Cyto: cytoplasmic

We examined IgG subclasses of ANA in systemic autoimmune diseases such as SLE, SSc, SS, and PM (Table 2). The ANA titers ranged from 1:40 to 1:5120 with various patterns. The Subclass-based ANA test detected IgG1^+^, IgG2^+^, or IgG3^+^ ANA in the systemic autoimmune disease cases (Fig. 2, 3). Especially, all cases were IgG2^+^. However, IgG4 was not detected (Fig. 2, 3), except in a patient with SS who showed IgG4-type ANA with peripheral pattern (Fig. 4).

**Table 2:** ANA profiles of patients with systemic autoimmune diseases

Case	ANA	Specific autoantibodies	IgG4^a^	IgG^a^
SLE 1^b^	Spe 320	dsDNA, ssDNA, U1-RNP, Sm	21.3	1830
SLE 2	Homo + Spe 320	dsDNA, ssDNA, SS-A	11	826
SLE 3	Spe 1280	dsDNA, ssDNA, Sm, Ribosome	20	2043
SLE 4	Spe 640	ssDNA, U1-RNP, Sm, SS-A, SS-B	8.3	829
SLE 5	Homo + Spe 1280	dsDNA, ssDNA, U1-RNP, Sm, SS-A, SS-B	7	556
SLE 6	Homo + Spe 160	ssDNA	48.6	1938
SLE 7	Spe 320	dsDNA, SS-A	19.6	1186
SLE 8	Spe 5120	dsDNA, ssDNA, U1-RNP, Sm, SS-A	7	908
SSc 1	Discrete spe 1280	Centromere	7	1177
SSc 2^b^	Discrete spe 1280, Spe 160, Cyto 80	Centromere, SS-A	21.2	1772
SSc 3	Spe 1280	Scl-70, U1-RNP, SS-A	25.5	2147
SSc 4	Discrete spe 1280	Centromere, Scl-70, U1-RNP	12.4	1108
SS 1	Spe 320	SS-A, SS-B	33.4	2974
SS 2	Spe 160	SS-A, SS-B	16.5	1765
SS 3	Spe 80	SS-A, SS-B	74	1370
SS 4^c^	Spe 640	SS-A	228	1721
SS 5	Spe 40, Cyto 80	SS-A	38	2133
SS 6	Spe 160	SS-A, SS-B	14.5	2340
SS 7	Spe 160	SS-A, SS-B	9.5	1882
SS 8^b^	Spe + Nucleolar 80, Cyto 40	SS-A	20.1	1678
PM 1	Spe + Nucleolar 640	Ku	53.5	1668
PM 2^b^	Spe 320, Cyto 40	ssDNA, U1-RNP, Sm, SS-A	12.9	1132
PM 3	Spe 40, Cyto 160	PL-7	15	717
PM 4	Spe 320	U1-RNP, Sm	5	282
PM 5	Spe 1280	Ku, SS-A, SS-B	<3	823
PM 6	Spe 40, Cyto 80	SRP	18.4	1365
PM 7	Homo + Spe 160	Not detected	19	2051

^a^mg/dL in serum. ^b^Shown in Fig. 3. ^c^Shown in Fig. 4

ANA: anti-nuclear antibody; Cyto: cytoplasmic; Discrete spe: discrete speckled; Homo: homogeneous; RNP: ribonucleoprotein; Spe: speckled; SRP: signal recognition particle

### An exceptional case with IgG4-type ANA

A 79-year-old male with SS showed IgG4-type ANA with peripheral pattern (Fig. 4). Lip biopsy results were compatible with SS, although anti-IgG4 staining was not performed. Anti-SS-A/Ro antibody was positive. We saw no swelling of lacrimal glands, salivary glands, or lymph nodes. This patient did not meet the criteria for IgG4-RD and was not considered to have clinical IgG4-RD.

### Difference of ANA patterns among IgG subclasses

In serum from one patient, ANA patterns differed among IgG subclass. Such phenomenon was seen in Fig. 3 and 4.

## Discussion

In IgG4-RD patients, we found no IgG4^+^ ANA, but did detect IgG1^+^ and IgG2^+^ ANA (Fig. 1 and 2). We also found IgG4^+^ ANA was very rare, whereas IgG1/2/3^+^ ANA were detected in systemic autoimmune diseases (Fig. 2 and 3). Autoantibodies with cytoplasmic patterns in the Fluoro-HepANA™ test are not exact ANA; “anti-cytoplasmic” antibodies—e.g., anti-SS-A/Ro, anti-aminoacyl-tRNA synthetase, and anti-signal recognition particle antibodies—are known in SS and PM. Subclass-based ANA tests found IgG1/2/3^+^ anti-cytoplasmic antibodies, but not IgG4 (Fig. 2, 3).

IgG4^+^ ANA is very rare in systemic autoimmune diseases, possibly because serum IgG4/IgG ratios are low, less than 5 %, in these diseases (Table 2). However, IgG4^+^ ANA was not detected despite high serum IgG4/IgG ratios (43 %) in IgG4-RD. This implies that IgG4 itself is not used to make ANA.

Several studies have investigated ANA subclasses in systemic autoimmune diseases. Zouali et al. reported that in SLE and mixed connective tissue disease, anti-double-stranded DNA (dsDNA) antibody was IgG1/3-dominant, and anti-RNP was IgG2-dominant [11]. Anti-Sm, anti-RNP, and anti-dsDNA in SLE [12], anti-SS-A/Ro and anti-SS-B/La in SS [13], and anti-Scl-70 in SSc [14] are reportedly IgG1-dominant. However, IgG4-type ANA was hardly detected in all the above reports. Rigopoulou et al. examined primary biliary cirrhosis cases, and found that ANA was IgG1/3-dominant but IgG4 was not detected by subclass-based IIF [15]. The reason IgG2-type ANA was remarkably frequent in our study whereas IgG1 and IgG3 were predominant in previous studies might be that second antibody affinities differed between studies. In the subclass-based ANA test, titers cannot be accurately compared between subclasses, as the second antibodies are different. In past studies, IgG2-type ANA was also detected at moderate levels, whereas IgG4-type ANA was constantly negative or at low levels. In our study, IgG4-type ANA was also hardly detected.

Autoimmune pancreatitis (AIP) is an organ-specific disorder seen in IgG4-RD. Various autoantibodies, such as anti-lactoferrin [5] and anti-carbonic anhydrase II [6] antibodies, are seen in AIP. Asada et al. found anti-pancreatic secretory trypsin inhibitor (PSTI) antibody in AIP, and showed that the titers of anti-PSTI antibody moved in parallel with serum IgG4 levels [16]. IgG4 levels change in parallel with IgG4-RD disease activity, as reported in many studies, including our previous study [17]. Asada et al. thought that anti-PSTI might be an important factor in the pathophysiology. However, immunoblotting of subclasses with anti-IgG1 or anti-IgG4 as second antibodies showed the subclass was not IgG4 but IgG1. Possibly, IgG4-type autoantibodies are difficult to produce in IgG4-RD patients.

However, some autoimmune diseases reportedly show IgG4-type autoantibodies. Rock et al. reported that IgG4 was the most common (100 %) of anti-desmoglein (Dsg)-1 antibodies detected in sera of patients with pemphigus foliaceus, and showed the pathogenicity of IgG4-type anti-Dsg-1 antibody using Balb/c mice [18]. Anti-Dsg-3 antibody in pemphigus vulgaris was also IgG4-predominant [19]. Beck et al. showed by immunoblotting that anti-phospholipase A_2_ receptor (PLA_2_R) antibody in idiopathic membranous nephropathy mainly consisted of IgG4 [20]. IgG4 is reportedly predominant in anti-neutrophil cytoplasmic antibody (ANCA). C-ANCA (IIF), proteinase-3 (PR3)-ANCA (ELISA), and myeloperoxidase (MPO)-ANCA (ELISA) in granulomatosis with polyangiitis (GPA) [21], and MPO-ANCA (ELISA) in propylthiouracil-induced vasculitis [22] were IgG1/4-dominant. Others similarly reported that IgG4 made up most C-ANCA (IIF) and PR3-ANCA (ELISA) in vasculitides [23, 24]. Engelmann et al. reported that anti-cyclic citrullinated peptide (CCP) antibody was IgG1/4-dominant in RA [25]. However, IgG4 in vasculitides and RA might not be pathophysiologically important. In functional analyses of ANCA, IgG1 and IgG3 PR3-ANCA can stimulate neutrophils [26], whereas IgG4 PR3-ANCA was only weakly stimulatory to neutrophils [27]. In RA patients who had HLA-DR4-shared epitope, Engelmann et al. found IgG3 anti-CCP antibody to be predominant, and considered that IgG3-type antibody might be more important in the pathophysiology of RA [28]. As IgG4 has poor ability to activate complements and antibody-dependent cellular cytotoxicity [29–32], IgG4 is unlikely to take part in mechanisms of tissue damage in autoimmune diseases.

Interestingly, there seem to be pathogenic and non-functional IgG4-type autoantibodies. IgG4-type ANCA is considered less pathogenic, compared to other subclass ANCA in ANCA-associated vasculitis [26, 27]. The affinities between IgG4-type and other subclass ANCA should be equal, but the abilities of complement activation are different, so that the role of IgG4-type ANCA can be less significant than that of other subclass ANCA. On the other hand, IgG4 anti-PLA_2_R antibody has high affinity and is considered pathogenic in idiopathic membranous nephropathy [20]. Why IgG4 anti-PLA_2_R antibody can exert pathogenicity without ability of complement activation may be because the pathogenicity is brought by the destruction of electrical barriers of glomerular basement membrane.

Taken together, IgG4 usage rates differ among autoantibodies and among diseases. IgG4 is associated with anti-Dsg-1/3, anti-PLA_2_R, anti-CCP antibodies, and ANCA, but not with anti-PTSI antibody in AIP or ANA in IgG4-RD and systemic autoimmune diseases (Table 3). This asymmetry implies that IgG4 has unknown but certain physiological or pathological functions. Further analyses are needed to know its role.

**Table 3:** Summary of predominant subclasses in autoantibodies in IgG4-RD and autoimmune diseases

Diseases	Autoantibodies	Predominant subclass	IgG4 subclass	Reports
IgG4-RD	ANA	IgG2	Negative	Present study
IgG4-RD (AIP)	Anti-PSTI	IgG1	Negative	Asada [16]
SLE, SSc, SS, PM	ANA	IgG1/2/3	Seldom	Present study, Zouali [11], Eisenberg [12], Maran [13], Vazquez-abad [14]
GPA, Vasculitis	ANCA	IgG1, IgG4	Frequent	Brouwer [21], Mellbye [23], Liu [24], Gao [22]
RA	ACPA	IgG1, IgG4	Frequent	Engelmann [25]
PF, PV	Anti-Dsg-1/3	IgG4	Primary	Rock [18], Ding [19]
Idiopathic MN	Anti-PLA_2_R	IgG4	Primary	Beck [20]

ACPA: anti-citrullinated protein antibody; AIP: autoimmune pancreatitis; ANA: anti-nuclear antibody; ANCA: anti-neutrophil cytoplasmic antibody; Dsg-1/3: desmoglein-1 and 3; GPA: granulomatosis with polyangiitis; MN: membranous nephropathy; PF: pemphigus foliaceus; PLA_2_R: phospholipase A_2_ receptor; PSTI: pancreatic secretory trypsin inhibitor; PV: pemphigus vulgaris; RA: rheumatoid arthritis

In the present study, we observed ANA patterns differed among IgG subclasses in some cases (Fig. 3, 4). When a case has several autoantibodies, the utilized subclasses differ by autoantigens. This can be explained by the hypothesis that each IgG subclass prefers to cover its own spectrum of antigens. The reason we hardly found IgG4 in ANA might be that IgG4 does not cover antigens that can be detected by the ANA test—i.e., nuclear antigens or related microbial antigens. Selective IgG2 subclass deficiency is often associated with bacterial infection by *Neisseria meningitidis* and *Streptococcus pneumoniae* [33, 34], so that IgG2 is considered to have a role in protection from these bacteria. The role of IgG4 has not been sufficiently understood. If IgG4 is related to some microorganism type, and if the microorganism antigens and autoantigens are similar, as with Dsg-1/3, PLA_2_R, PR3, and citrullinated proteins, it would explain why IgG4-type antibody against those proteins was dominantly generated.

Our results imply that IgG4-RD is not an autoimmune disease, and that high levels of serum IgG4 in IgG4-RD are only nonspecific. Subclass-based ANA tests in this study covered both nuclear and cytoplasmic antigens in HEp-2 cells, and can screen a wide range of unmodified ubiquitous antigens. However, this analysis has limitations: modified antigens like citrullinated proteins and organ-specific antigens are not screened. The number of cases is limited in this study. There remains a possibility that unknown IgG4-type autoantibodies might be found in IgG4-RD. A further analysis is needed.

## Conclusions

We found ANA in IgG4-RD patients are not IgG4-based despite high serum IgG4 levels. IgG4 was also hardly found in ANA in systemic autoimmune diseases. We also observed several patients in whom ANA patterns differed among IgG subclasses, probably due to difference in corresponding autoantigens. These findings imply that each IgG subclass tends to cover its own spectrum of antigens, and IgG4 is not apparently used to make ANA.

## Abbreviations

ANA: Anti-nuclear antibody
ANCA: Anti-neutrophil cytoplasmic antibody
AIP: Autoimmune pancreatitis
CCP: Cyclic citrullinated peptides
dsDNA: Double-stranded deoxyribonucleic acid
Dsg: Desmoglein
ELISA: Enzyme-linked immunosorbent assay
GPA: Granulomatosis with polyangiitis
HLA: Human leukocyte antigen
IgG4-RD: Immunoglobulin G4-related disease
IIF: Indirect immunofluorescence
MPO: Myeloperoxidase
PLA_2_R: Phospholipase A_2_ receptor
PM: Polymyositis
PR3: Proteinase-3
PTSI: Pancreatic secretory trypsin inhibitor
RA: Rheumatoid arthritis
RNP: ribonucleoprotein
SLE: Systemic lupus erythematosus
SS: Sjögren’s syndrome
SSc: Systemic sclerosis.
